# Advanced Microfluidic Technologies for Lipid Nano-Microsystems from Synthesis to Biological Application

**DOI:** 10.3390/pharmaceutics14010141

**Published:** 2022-01-07

**Authors:** Bruna G. Carvalho, Bruno T. Ceccato, Mariano Michelon, Sang W. Han, Lucimara G. de la Torre

**Affiliations:** 1 Department of Material and Bioprocess Engineering, School of Chemical Engineering, University of Campinas (UNICAMP), Campinas 13083-852, Brazil; gregatti.bruna@gmail.com (B.G.C.); b264181@dac.unicamp.br (B.T.C.); 2 School of Chemical and Food Engineering, Federal University of Rio Grande (FURG), Rio Grande 96203-900, Brazil; michelon@furg.br; 3 Center for Cell Therapy and Molecular, Department of Biophysics, Federal University of São Paulo (UNIFESP), São Paulo 04044-010, Brazil; sang.han@unifesp.br

**Keywords:** microfluidics, lipid nanoparticles, liposome, artificial lipid cells, gene delivery, drug delivery

## Abstract

Microfluidics is an emerging technology that can be employed as a powerful tool for designing lipid nano-microsized structures for biological applications. Those lipid structures can be used as carrying vehicles for a wide range of drugs and genetic materials. Microfluidic technology also allows the design of sustainable processes with less financial demand, while it can be scaled up using parallelization to increase production. From this perspective, this article reviews the recent advances in the synthesis of lipid-based nanostructures through microfluidics (liposomes, lipoplexes, lipid nanoparticles, core-shell nanoparticles, and biomimetic nanovesicles). Besides that, this review describes the recent microfluidic approaches to produce lipid micro-sized structures as giant unilamellar vesicles. New strategies are also described for the controlled release of the lipid payloads using microgels and droplet-based microfluidics. To address the importance of microfluidics for lipid-nanoparticle screening, an overview of how microfluidic systems can be used to mimic the cellular environment is also presented. Future trends and perspectives in designing novel nano and micro scales are also discussed herein.

## 1. Introduction

The development of lipid nano-microsystems has initiated in the middle of the last century with Alec Bangham. He developed the thin-film method in a laboratory protocol to produce lipid structures that mimic the lipid bilayer [1]. These structures were named liposomes and have been extensively studied as membrane models.

Liposomes are self-aggregated colloidal systems in a bilayer structure composed of phospholipids, forming a spherical vesicle with an aqueous core [2]. Conventional liposomes can be large unilamellar vesicles (LUV), sizes ranging between 100–1000 nm, or small unilamellar vesicles (SUV), from 20 to 100 nm. Another size classification is giant unilamellar vesicles (GUV)—microstructures larger than 1000 nm—which are usually referred to as giant liposomes [3,4]. Liposomes are formed through self-assembly processes caused by unfavorable interactions between phospholipids and water, related to an increase in medium polarity [5,6]. Liposomal systems can be used for nano-encapsulation of drugs and bioactive molecules for diverse applications in the biomedical and medical fields [2,7,8].

Later, at the end of the last century, liposomes were investigated as drug delivery systems due to their amphiphilic nature, allowing the incorporation and conventional encapsulation of different drugs. Doxil^®^ (Alza Corporation, Mountain View, CA, USA) was the first U.S. Food and Drug Administration (FDA)-approved PEGylated nano-liposomes [9] to treat ovarian cancer and AIDS-related Kaposi’s sarcoma [10]. Since its approval in 1995, the investigation of liposomes has extensively increased [11,12]. In parallel, since the 1990s, when the first concepts of clinical gene therapy were well-established, the intention of effectively treating and preventing diseases relying on gene information became a possible and promising reality [13]. Gene therapy is based on the intracellular delivery of therapeutic nucleic acids to correct inherited genetic disorders or combat acquired diseases [14,15]. The therapeutic RNA or DNA has to be internalized and released within the cells for proper therapeutic effect. From this perspective, non-viral vehicles became an attractive means of transporting nucleic acids due to long experience with liposomes and greatly reduced biosafety issues [16,17]. These complexed nanoparticles are named lipoplexes or lipid nanoparticles (LNPs). These nanocarriers generally explore the difference between the anionic genetic material charge and cationic charge of lipids. By doing so, a high encapsulation efficiency, reduced immune responses, and ease of fabrication can be achieved [18,19].

After the approval of Onpattro^®^ (Alnylam Pharmaceutics, Cambridge, MA, USA) in 2018 by the FDA, which is small interfering RNA (siRNA) LNPs for amyloidosis treatment, LNPs have been extensively studied as potential gene carriers for vaccines. In this sense, it is possible to highlight the recently approved Pfizer COVID-19 vaccine in the US [20,21,22]. Even though these nanocomplexes have many advantages, as mentioned before, one limiting factor for using these systems relies on the synthesis process. The conventional methods are generally discontinuous and use the top–down approaches, requiring post-processing steps to decrease the average size and polydispersity. Besides, traditional techniques present limitations in terms of suitability for scaling up from the bench-scale to the industrial production; it happens because size distribution shows low reproducibility from batch to batch [23]. In this context, at the end of the last century, microfluidic technology was investigated to produce liposomes in a bottom-up approach.

Microfluidics is defined as the science and technology that investigate and/or apply the study of behaviors of fluids, controlled fluid manipulations, and the design of such devices or systems that can reliably perform such tasks in microchannels with typical dimensions of micrometers [24,25]. Commonly, microfluidic devices have sizes ranging from a few millimeters to micrometers, and they are characterized by exhibiting at least one channel with a dimension smaller than 1 mm [26,27]. The main advantages of microfluidics for lipid nano-microsystems synthesis include the use of strictly laminar flow, short molecular diffusion distances, large specific surface areas, heat transfer characteristics, low energy consumption, and precise flow control.

**Figure 1:**
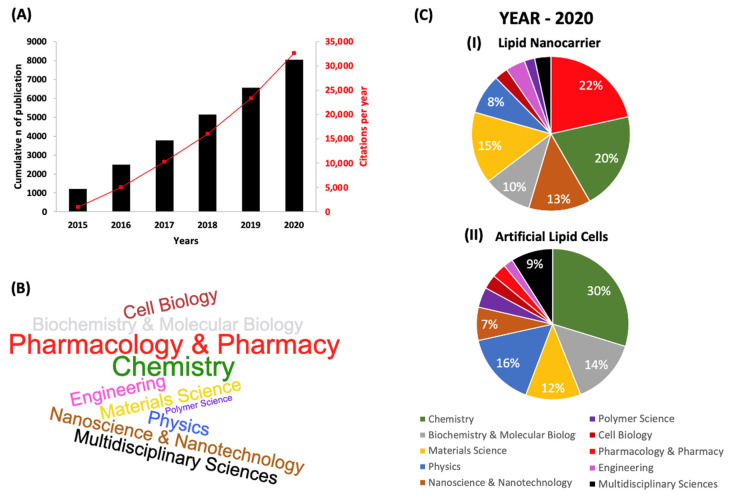
(**A**) Microfluidic publications over the last five years concerning studies with “Microfluidic*” as a search term along with “Liposome”, “Lipid-nanoparticle”, “Giant Lipid Vesicle or Giant Liposome”, and “Artificial Lipid Cells”. The keywords were searched for on the Web of Science (WoS) analytics report system from Thomson Reuters. The symbol * takes into account permutations of the keyword. (**B**) Word cloud illustrates the most frequently used microfluidics for the synthesis or application of lipid nano-microsystems. The font size is proportional to the number of publications in 2020. (**C**) Pie charts describing the number of microfluidic publications in various disciplines related to (**I**) lipid nanocarriers and (**II**) artificial cells (in 2020). The literature search was performed using WoS to determine the number of microfluidics publications.

The most significant advantage of synthesis processes using microfluidics relies on developing sustainable technologies with less financial demand. Microchannels also can be parallelized for amplification purposes and increased production. This fact reduces costs and time for translation technology from bench to bedside [27]. According to the projections, the global market for microfluidic technologies reached USD 6.8 billion in 2017 and should reach nearly USD 13.9 billion by 2022 [28]. Recently, microfluidics has been reported as a century technology [25,29,30]. Especially in the nano-micro lipid system field, there was a rapid development in the last years, which can be confirmed by the significant increase in publications (Figure 1) in pharmacology and pharmacy.

**Figure 2:**
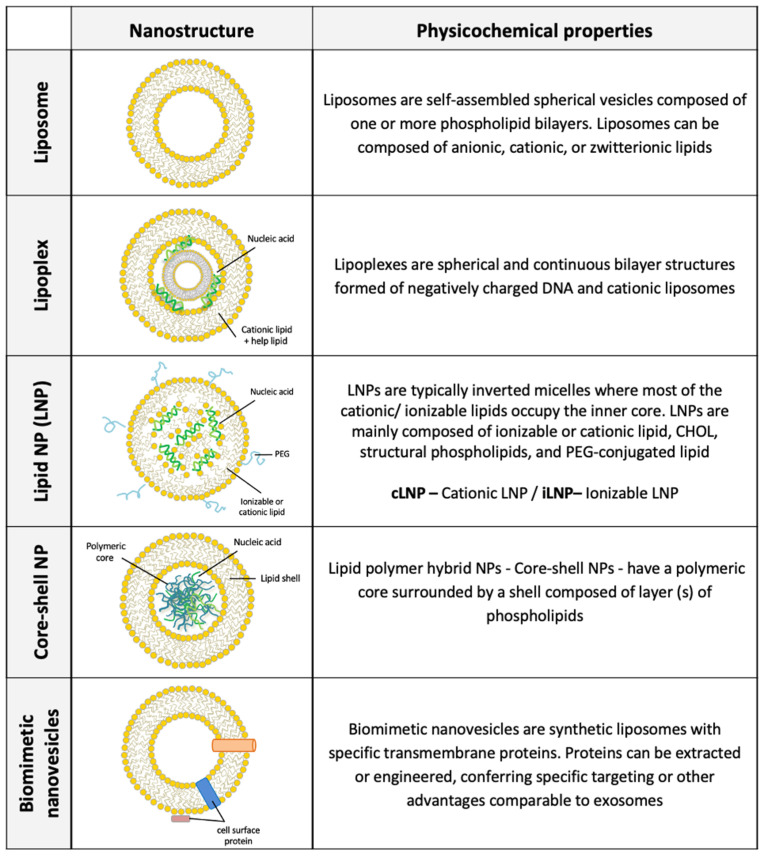
The main characteristics of lipid-based nanocarriers used for drug and gene delivery (liposomes, lipoplexes, lipid nanoparticles (LNP)—cLNP and iLNP—core-shell NP, and biomimetic nanovesicles). cLNP:LNP with cationic lipids (non-ionizable lipids) such as DOTAP. iLNP:LNP with a cationic source is the ionizable lipids.

In the field of pharmacology and pharmacy, there is a myriad of applications using lipids and microfluidic technology, as lipid nano-micro sized structures for drug and gene delivery and revolutionary platforms for lipid-based nanocarrier assays. This review will describe the recent advances in the production of lipid nanostructures and their application. The main characteristics of lipid nanostructures discussed in this review are illustrated in Figure 2.

This paper also introduces the microfluidic approaches to synthesize lipid micro-sized structures, as GUVs, and new strategies for the controlled release of lipid nanostructures from microgels produced by droplet-based microfluidics. This review will also give some insights into recent trends on microfluidic platforms for lipid nanoparticle screening and some brief and relevant information about the main manufacturing techniques and materials regarding lipid nano and microstructures synthesis.

### 1.1. Overview of Microfluidic Techniques for Lipid Nano-Micro Sized Structures

There are mainly two methods for producing lipid nanoparticles: top-down and bottom-up processes [31,32]. Top-down approaches, such as thin-film hydration and ethanol injection, are commonly named bulk methods. They often need non-standard multistep procedures to reduce the particles’ size and polydispersity. These batch methods are usually coupled with size-reduction steps, such as sonication or extrusion. To overcome these drawbacks, some continuous production techniques, such as microfluidics, have gained attention. Microfluidics manipulate fluids at the micro-scale, allowing continuous production using the bottom-up approach without size-reduction steps [31,33].

In most cases, microfluidics is based on a laminar flow regime, defined by a low Reynolds (Re) number [34,35]; see Box 1. Besides the advection (in a continuous flow), the mixing phenomenon is governed by diffusion. In microfluidics, nanoparticles can be synthesized with high uniformity and a suitable size for nano applications (around 50–300 nm), making it possible to control the flow and mixing conditions [36,37,38].

Box 1 **Dimensionless numbers and definition of physical terms used in microfluidics.**

Reynolds (Re) number: A dimensionless number that indicates the type of flow, laminar or turbulent, through the ratio between inertial and viscous forces, defined by the multiplication of the density of the fluid, characteristic length, and velocity, divided by the viscosity of the fluid.

Laminar regime: Fluid dynamics is characterized by the smooth flow. On a flat surface, the flow occurs in parallel lamellae. Low Re numbers describe it (typically Re < 2000), and the mixing process is governed by the phenomenon of molecular diffusion and characterized by highly predictable kinetics.

Chaotic advection: The mixture phenomenon where the advected particles suffer repeatedly transverse vortices, leading the fluids to wrap into one another, commonly called “stretching and folding”.

Regarding the laboratory scale, polydimethylsiloxane (PDMS) and soft lithography are the most common microdevice material and manufacturing techniques because of their low cost and being suitable for rapid prototyping [39,40,41]. For a scale-up and large production, strategies such as parallelization, modularization, and high flow rate microdevices are being developed to meet industrial requirements [32,42,43]. For more information, the main manufacturing devices techniques and materials used to synthesize lipid nano- and microstructures are presented in Box 2.

Box 2 **Manufacturing techniques and materials often used in microfluidics.**

Microfluidic devices materials: The basic manufacturing materials need to be the suitable with the most common fabrication techniques. The main materials include PDMS [40,44], polymethylmethacrylate (PMMA) [27,45], other polymers [41,46], glass [47], silicon [43,48], and paper [49,50].

Microfluidic devices manufacturing techniques: There are several techniques capable of manufacturing microdevices. It is worth mentioning photo [34,51] and soft [52,53] lithography, screen [54,55] and 3D printing [56,57], micromilling [8,58], xurography [59,60], etching [49,61], and micromachining and laser ablation [27,62]. More general information about the materials, techniques, and equipment commonly used in microfluidic manufacturing technology can be seen in Salwa and Kumar [39] and Lei [46]. For detailed information about the materials we highlight the work of Ren, Zhou and Wu [63], and that of Charmet et al. [59] regarding low-cost techniques.

**Figure 3:**
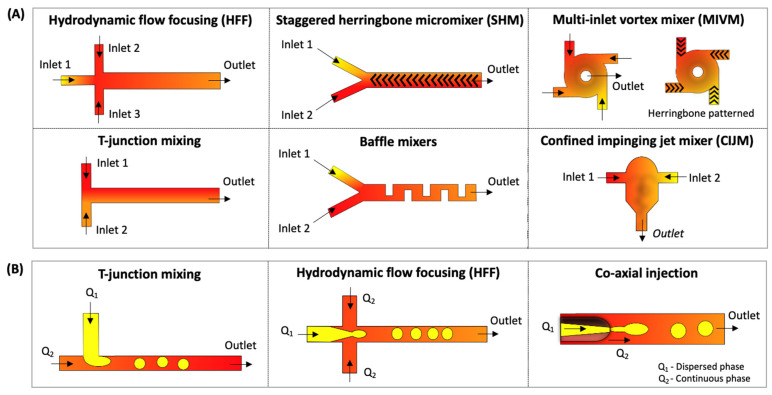
(**A**) Microfluidic techniques for drug and gene delivery nanoparticle formulation (liposome, lipid nanoparticles, and core/shell nanostructures). (**B**) Droplet-based microfluidics for lipid-based nanostructure encapsulation and artificial lipid cell synthesis.

In general, lipid nanostructures can be formed by mixing two inlet streams containing lipids in a water-soluble solvent and another central inlet with an aqueous solution. As the streams flow in parallel, the mixing process is initiated, and polarity changes, favoring the lipid autoaggregation in a reproducible manner [11,64]. The most applied microfluidic methods for producing liposomes include microfluidic hydrodynamic flow-focusing (HFF), chaotic advection-based micromixer (CA-M), see Box 1, such as staggered herringbone micromixer (SHM), and droplet-based microfluidics [65,66,67]. The main microfluidic geometries described in this review are presented in Figure 3.

#### 1.1.1. Microfluidic Hydrodynamic Flow-Focusing (HFF)

HFF can be mainly applied through 2D or 3D hydrodynamic flow-focusing (HFF) devices using three inlet streams with a cross-shaped geometry (Figure 3A). In this method, lipid nanostructures are formed at the stream interface due to an aqueous solution and ethanol (containing the lipids) diffusion, resulting in diffusive mixing and local dilution of the organic phase. This method is widely used to produce lipid nanostructures, mainly liposomes and lipoplexes. Despite producing small-sized lipid nanostructures with a narrow particle size distribution and easy to operate, the limited flow rates hinder a scale-up possibility and high throughput [11,38]. Besides synthesizing nanoparticles with more complex structures that need a high mixture level and encapsulation efficiency, such as the lipid nanoparticles (LNP), other techniques have proven more efficient. In this sense, the chaotic advection-based micromixers have emerged and been applied in the last five years to produce lipid nanostructures, such as liposomes [68,69] and LNPs [70,71,72].

#### 1.1.2. Microfluidic Chaotic Advection Micromixers (CA-M)

CA-M method was created to overcome the productivity limitation of HFF, allowing the production of lipid nanostructures at higher flow rates and improving the mixture efficiency [66,73]. Barriers inside the microchannel are introduced to generate chaotic advection, promoting the repetitive overlap of the streamflows. One example of a micromixer is the SHM (“herringbone grooves”); the required channel length for complete mixing can be reduced, usually using a two- or three-inlet device (Figure 3A) [74,75]. Even though SHM is considered a high-throughput production method and capable of scale-up by parallelization, it requires more expensive microfabrication processes due to its complex design [58]. Some drawbacks are the need for a controlled environment, such as a soft (or photo) lithography, which cannot provide a high-enough bonding strength caused by the sealing method for polydimethylsiloxane (PDMS)-based devices, limiting the operation at high flow rates [44,76]. As an alternative to SHM, other geometries that apply chaotic advection have emerged lately: a high flow rate microfluidic device (HFR-MD) with a 3D-twisted cross-sectional microchannel [77], toroidal mixer (TrM) for scale-independent production [32], spiral micromixer [78], and microfluidic oscillator mixer [79].

#### 1.1.3. Droplet-Based Microfluidics

This method produces highly monodisperse droplets using immiscible phase fluids (water/oil emulsions and liquid/gas). Droplet-based microfluidics may be used to produce micro-sized particles, such as GUVs and microgels [80,81]. In this sense, droplet microfluidics can be used to generate highly reproducible and homogenous microparticles. Figure 3B illustrates the main device geometries that can be used to form well-controlled interfaces and monodisperse emulsions, such as a T-shaped junction, HFF, and co-axial injection.

## 2. Microfluidics for Lipid Nano-Sized Structures Synthesis: Drug and Gene Delivery

Microfluidic research has been focused on producing efficient drug and gene carriers. The recent advances in medicine have driven discoveries of potential delivery carriers. The main types of nanocarriers synthesized by microfluidics will be discussed (Table 1 and Table 2).

### 2.1. Liposomes for Drug Delivery

A liposome’s amphiphilic nature allows the transportation of soluble and low-solubility compounds, which can be entrapped in the aqueous core and bilayer membranes. In general, the encapsulation of drugs into liposomes can be used to (i) control drug release and modify biodistribution; (ii) protect drugs from in vivo degradation; (iii) enhance solubility and bioavailability; and (iv) deliver to a specific tissue using specific ligands [106,107]. As an alternative to conventional methods, drug-encapsulated liposomes have also been produced via microfluidics. Microfluidics has emerged as a technique to merge liposome manufacturing and drug encapsulation in a single process step, leading to an overall reduced process time. Microfluidic-based drug-loaded liposomes have been used to deliver small molecules (hydrophobic and hydrophilic drugs) [82,83,108] and proteins [69,109]. Even though the entrapping of hydrophobic drugs is possible in liposomes, the encapsulation efficiency is typically low since they are only entrapped in the bilayer interface [68].

**Figure 4:**
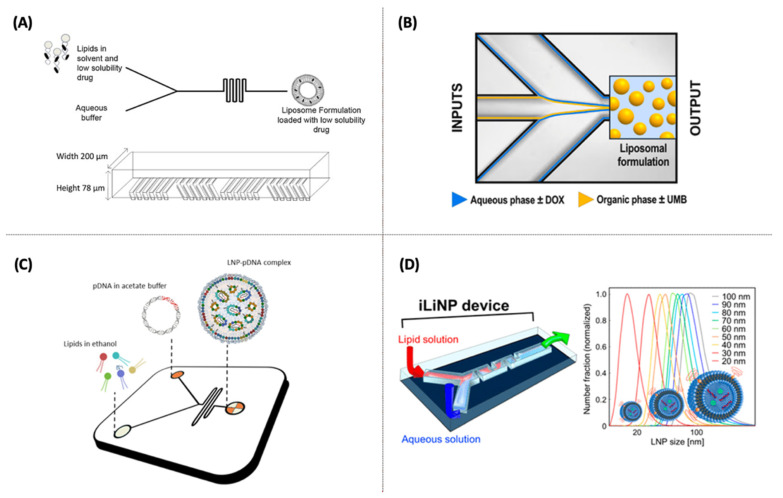
(**A**) Schematic depiction of the liposome formation process based on the SHM design, a chaotic advection micromixer for drug-loaded liposomes, and the chamber layout [68]. (**B**) Microfluidic system schematic (5-input chip mixing junction, Dolomite, Royston, UK), highlighting the mixing junction [85]. (**C**) Schematic of the lipid nanoparticle-plasmid DNA (LNP-pDNA) complex formulation strategy employing the Y-shape staggered herringbone micromixer (SHM) (NanoAssemblr Benchtop™, Vancouver, BC, CA) [89]. (**D**) Three-dimensional view of the invasive lipid nanoparticle (iLiNP) device and the size range of LNPs synthesized [90].

In this perspective, Kastner et al. [68] designed liposomes through a chaotic advection micromixer (SHM device) (see Figure 4A) for the solubilization of propofol, which is a poorly water-soluble drug. The high-throughput setting allowed the production of nanovesicles (from 50 to 450 nm) with a high drug loading (41 mol%). Compared to liposomes produced by thin film and sonication, microfluidic liposomes had significantly more drug encapsulated than sonicated liposomes (20% mol). Moreover, microfluidic liposomes showed good stability and remained unaffected after storage over eight weeks at 4 °C and 25 °C. The same research group encapsulated hydrophilic drugs (glipizide) and lipophilic drugs (metformin) in liposomes using an SHM setup. The authors entrapped drugs both individually and in combination. The authors found that in combination, it was possible to get better drug-loading efficiencies compared to the initial amount added, namely, 20–25% mol and 40–42% mol for the water-soluble and bilayer-embedded drugs, respectively. However, the co-loading impacts the drug release profiles up to 2 fold compared to liposomes containing a single drug alone [83].

In another study, SHM devices were used to produce a liposomal formulation for curcumin (CUR) entrapment, a hydrophobic drug, which can be used as chemosensitizers in cancer treatments. The authors noted that CUR-loaded liposomes given at a single and low dose exhibited a significant anticancer effect. Additionally, they found that the antitumor efficacy in tumor-bearing EMT6 and B16F10 mice could be increased by combining CUR-loaded liposomes with cisplatin (CIS) and alkylating antineoplastic drugs at a relatively low dose. The drug-loading efficiency found was around 17 wt%, a superior loading value (700-fold) than other liposomal systems reported in the literature. Besides that, the treatment with CUR reduced CIS nephrotoxicity [84].

Another potential application is shown by Gkionis et al. [85] (Figure 4B), who presented a liposomal formulation to co-load DOX and umbelliprenin (UMB), which is a natural compound with anti-inflammatory, anti-angiogenic, and anti-tumoral activity. The authors reported that DOX liposomes prepared either passively or actively exhibited an efficiency encapsulation of 81%, and the active loading of DOX and UMB were around 74% and 47%, respectively. When targeting breast cancer cells, MCF-7, MDA-MB231, and BT-473, DOX:UMB co-loaded liposomes showed lower toxicity than free DOX administration.

Controlling the pharmacokinetic drug distribution profile to improve therapeutic efficacy in liposome drug delivery systems is still a challenge. The pharmacokinetics and tissue distribution of the liposomes may affect their therapeutic effect and toxicity [106]. One alternative to deal with the drug accumulation in off-target cells is to design nanoparticles with specific ligands, such as folate, (Arg–Gly–Asp) tripeptide-RGD, cell-penetrating peptide TAT, and/or antibodies. This strategy may overcome toxicity issues while improving drug-delivery efficacy to specific cells. Within this perspective, Ran et al. [86] developed dual-ligand PEG-liposomes (folic acid and TAT) using a single-step microfluidic HFF device. This study showed that liposomes with both targeting ligands achieved better synergistic effects than single-ligand liposomes (TAT or folate liposomes) and PEG-liposomes. Enhanced results were found in vitro in SKOV3 and MCF-7 cells and 3D tumor spheroid models. An in vivo study using a xenograft model bearing a SKOV3 tumor further confirmed the improved tumor targeting and longer tumor retention (up to 72 h) of the dual-ligand liposomes.

Focus on translating liposomes from the bench to the clinic, Forbes et al. [69] used a microfluidic device to produce liposomes while incorporating insulin, bovine serum albumin (BSA), or ovalbumin (OVA). Additionally, the authors incorporated in the SHM device an in-line purification and at-line monitoring of the particle size for in-process control and a product validation tool in real time. The liposome formulations produced by the microfluidics offer a high protein loading (20–35%) compared to sonication or extrusion methods (<5%). It was only possible because using the in-line purification and at-line size monitoring step-up allowed the authors to optimize the best operating range for effective production of highly loaded liposomes with size control (60–100 nm) and a PDI < 0.2 high.

### 2.2. Cationic Liposomes (CLs) and Cationic LNPs for Gene Delivery

The proper delivery of genetic material to cells can be difficult due to several hurdles. In general, naked nucleic acids delivery (DNAs and RNAs) are susceptible to rapid elimination from circulation due to several factors, such as biological barriers (nucleases and intracellular compartments), the size, and the negative charge of the genetic material [18,110]. The electrostatic complexation with cationic lipids was one of the initial strategies to protect and complex the genetic material, which complexes the negatively charged genetic material. Cationic and ionizable lipids are chemically synthesized, and different molecules have been investigated over the years [107]. Preformed CLs can also be used with different compositions to complex the genetic material, generating lipoplexes. Later, LNP emerged from the prior knowledge of lipoplexes (Table 1).

LNPs follow the non-bilayer theory and are not designed to have multilamellar structures, which induces the proper release of genetic material inside cells and increases the biological response. This feature is modulated by changing the lipid composition, which is carried out in a one-step synthesis [111,112,113,114]. Nowadays, LNPs have become one of the leading gene-delivery nanocarriers [22,115].

### 2.3. pDNA-Based Non-Viral Lipid Vectors

A typical plasmid DNA (pDNA) needs to be substantially condensed to achieve a suitable size for proper delivery, which relies on about 100 nm or less sizes. To encode the therapeutic protein, DNA has to reach the cell nucleus to allow access to the transcriptional machinery [18,110].

To deliver pDNA safely, Balbino et al. [87] produced lipoplexes (pEGFP-N1)—pDNA carrying enhanced green fluorescent protein (cDNA)—in one-step using multiple HFF regions. The microfluidic platform was designed first to synthesize liposomes and then to condense them with pDNA. The lipoplexes achieved similar transfection efficiencies as lipoplexes prepared by conventional bulk processes, around 40%, proving the process feasibility to produce nanoliposomes. The same authors found a similar transfection efficiency when synthesizing pEGFP-N1/CLs in an HFF microdevice with pDNA being hydrodynamically compressed by two CLs side streams [88].

Kulkarni et al. [92] studied several designs of LNPs for delivery of pDNA carrying EGFP or firefly luciferase (FLuc) to explore how the lipid composition can affect the transfection efficiency in mammalian cell lines and primary cells. Synthetized through a T-junction micromixer, the LNPs containing ionizable amino lipids proved to be a highly effective and non-toxic delivery system for pDNA, both in vitro and in vivo, with green fluorescent protein (GFP) expression above 85%. Mucker et al. [93] used a Nanoassemblr^®^ (Precision Nanosystems, Vancouver, BC, Canada) microfluidic device to incorporate pDNA pWRG/c7d11 in LNPs. The results showed that LNPs could successfully be applied as DNA prophylactic Andes and Zika virus DNA vaccines capable of producing elevated neutralizing antibodies in rabbits and nonhuman primates.

To generate pDNA (GL3)-LNPs, Quagliarini et al. [89] used a Y-shape SHM with two inlets (Figure 4C) to investigate the transfection efficiency of pDNA-loaded LNPs. It was seen that PEGylation and sample concentration were essential to obtain homogeneous and small-size LNPs with a high transfection efficiency and minor cytotoxicity in HEK-293 cells. The total flow rate also proved to affect both the physicochemical properties and consequently the transfection levels of LNPs. The work addressed significant gaps left in the literature about pDNA-loaded LNPs, which are less explored, so far, than RNA-loaded LNPs, as discussed by the authors.

Applying a T-junction mixer, Kulkarni et al. [94] analyzed the mixing of mRNA FLuc and pDNA encoding TdTomato with ionizable amino-lipids mixing to produce LNPs. The results showed that the nucleic acid size influences the LNP size distribution; for instance, mRNA, minicircle DNA (mcDNA), and pDNA are more likely to lead to two population formation, loaded LNPs, and “empty” LNPs. Analog results were reported by Roces et al. [70], in that the morphology of LNPs synthesized using Y-shape SHM depend on the nucleic acid size. The authors reported that LNPs produced with polyadenylic acid (PolyA), single-stranded deoxyribonucleic acid (ssDNA), and mRNA Fluc had different final morphological characteristics. Besides, the PDI across all formulations tested was below 0.25, and high encapsulation efficiencies were achieved for all the LNP systems (>90%).

Although DNA-LNPs are gaining attention, the necessity to cross the membrane barrier of the nucleus invigorates the advantages of RNA-LNPs [22].

### 2.4. RNA-Based Non-Viral Lipid Vectors

The main difference between RNA and DNA delivery is related to the intracellular local delivery site. The RNAs mostly used for therapies, such as messenger RNA (mRNA), silencing RNA (siRNA), microRNA (miRNA), and self-amplifying mRNA (SAM), only need to reach the cytoplasm to promote protein expression or inhibition [18,110,116].

Despite being a simple molecule structure, the non-replicating messenger RNA (mRNA) presents limited in vivo stability and activity due to the limited duration of protein expression inside cells. Possible toxicity also can arise from the protein expression at off-target sites, leading to unwanted protein expression [117,118]. Hence, chemical modifications can tune RNA delivery and increase protein expression and its activity [22,119]. In this sense, SAM can induce prolonged local protein expression and its activity with lower doses than conventional mRNA [120,121]. Once in the cytoplasm, SAM functions as a translation template to produce the RNA-dependent RNA polymerase and then make multiple identical copies of the original RNA strand [115,122].

siRNA and miRNA are double-stranded RNA molecules that silence target genes via RNA interference (RNAi) and enable specific silencing of virtually any gene in the human genome [123]. After reaching the cytoplasm, siRNA and miRNA interacts with the RNA-induced silencing complex (RISC), and then, the RISC is guided to the target mRNA, which is recognized and cleaved or blocked for translation [66,124]. As these RNAs are much smaller than mRNA and pDNA, they can enter the cell easier.

The capacity of siRNA to silence hepatic genes in vivo has been well-established since the approval of Onpattro^®^(Alnylam Pharmaceutics, Cambridge, USA). This effect is due to the ability of ionizable LNPs to adsorb apolipoprotein E in the circulation, giving rise to a natural targeting ligand that binds with high affinity to the low-density lipoprotein receptor that facilitates the release of siRNA into the cytoplasm by endocytosis [118,125]. So far, several LNPs have been developed by microfluidics to deliver siRNA [126,127,128,129]. Kimura et al. [90] synthesized different sizes of LNPs (20–100 nm) to potentialize the siRNA plasma coagulation factor VII (siRNA FVII). The LNPs were effectively delivered in vivo to hepatocytes of the extravascular region, using an invasive lipid nanoparticle production device (iLiNP), for which the effectiveness of the mixing was compared with SHM. The device with baffle mixer structure has a simple two-dimensional microchannel, as shown in Figure 4D, and proved to have better LNP size controllability and productivity over conventional SHM.

Another type of therapeutic RNA that has gained attention using microfluidic technology for gene therapy is mRNA [130,131]. Patel el al. [91] used microfluidic mixing to produce Fluc, EGFP, and mCherry mRNA LNPs, and compared them for gene transfer applied to the back of the eye. The authors observed that LNPs containing ionizable lipids with low pKa, such as Dlin-MC3-DMA (MC3) and Dlin-KC2-DMA (KC2), showed the most significant amount of reporter gene transfection in the retina after subretinal injection.

All the advantages in RNA-like LNPs show massive research on technologies to improve RNA-LNPs, which leads to more vaccines and therapeutic candidates in clinical trials [111,113,115]. In this sense, microfluidics plays an important role, as it is the main technology to synthesize these nanoparticles, and consequently, it evolves at the same speed.

### 2.5. Lipid-Polymer Hybrid (Core/Shell) Nanoparticles Synthesis for Drug/Gene Delivery

Over the years, the focus of nanoparticle design has evolved toward complex single delivery systems that combine multiple functionalities within the same nanoscopic architecture. These hybrid structures, such as core-shell lipid-polymer hybrid nanoparticles, have emerged as a robust and promising delivery platform. These integrated systems, also known as core-shell nanoparticles, have been introduced to mitigate some limitations associated with liposomes and biodegradable polymeric nanoparticles. The lipid shell presence overcomes the main limitations of polymeric nanoparticles, such as the burst release and reticuloendothelial absorption [132,133,134]. Core-shell nanostructures combine the mechanical advantages of biodegradable polymeric nanoparticles and the biomimetic advantages of liposomes. These hybrid architectures may provide some advantages, such as surface functionality, high drug loading, entrapment of multiple therapeutic agents, tunable drug release profile, and good serum stability [132].

From this perspective, microfluidics has gained substantial attention to prepare hybrid nanoparticles as an alternative to the one-step bulk method. Typically, the convectional one-bulk approach combines single-step nanoprecipitation and self-assembly processes. Thus, this review section focuses on the current research trends on core-shell nanoparticles produced by microfluidics applied for drug and gene delivery.

**Figure 5:**
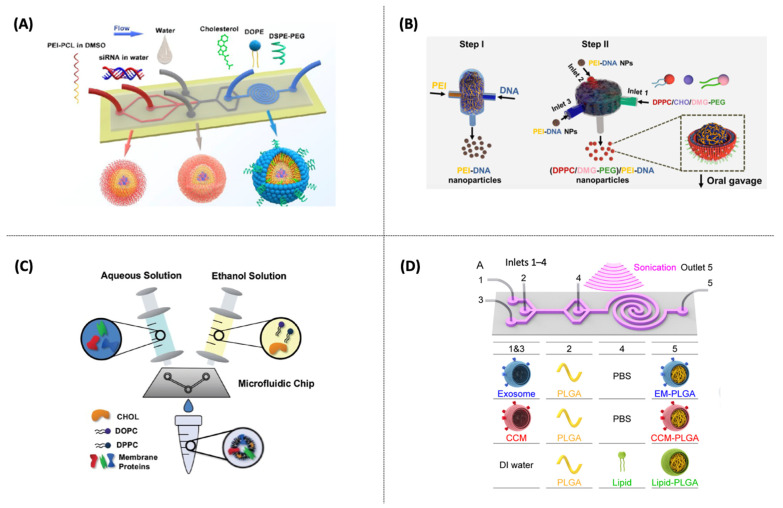
(**A**) Preparation of core-shell lipid/PCL-PEI/siRNA with the aid of a three-stage microfluidic device [95]. (**B**) Schematic illustration of core-shell lipid/PEI-DNA nanoparticle preparation via flash nanocomplexation (FCN) using a confined impinging jet device (CIJ) and multi-inlet vortex mixer (MIVM) [96]. (**C**) Microfluidic HFF system used to produce biomimetic nanovesicles (Leukosomes) [102]. (**D**) Schematic of one-step microfluidic sonication method to assemble biomimetic core—shell NPs (exosome membrane (EM), cancer cell membrane (CCM), and lipid—coated PLGA NPs) [103].

Microfluidic-based core-shell nanoparticles have delivered different nucleic acids such as siRNA, mRNA, and pDNA [95,96,97]. Wei et al. [95] designed a novel lipid/polymer hybrid nano assembly (see Figure 5A) composed of siRNAs complexed in the inner hydrophilic core of reverse PCL-PEI micelles followed by coating a neutral lipid membrane. Compared to lipid/micelle/siRNA nanoparticles prepared with a bulk mixing method, the core-shell nanostructure produced via microfluidics exhibited more robust protection of siRNA locked in the core and better stability in circulation. Moreover, microfluidic-based nanoparticles showed significant downregulation of EGFR (epidermal growth factor receptor) mRNA and protein expression levels in vitro and in vivo and significant tumor growth inhibition.

Even though microfluidic-based lipid/polymer hybrid nano assemblies demonstrated promising clinical application delivery results, achieving the biomanufacturing requirements is still a challenge. Many current nanoformulation methods, such as bulk mixing and hydrodynamic focusing, still require continuous, scalable, and reproducible technologies to increase their limited yield. In this context, robust self-assembly technologies that decrease heterogeneity and batch-to-batch variation are still needed. In this perspective, flash technologies facilitate nanoparticles’ self-assembly and formulation in a low-cost, high-throughput, and controllable manner. Flash nano complexation (FCN) and flash nanoprecipitation (FCP) involve rapid mixing in confined impingement jets mixers (CIJM) or multiple inlet vortex mixers (MIVM) [135] (Figure 3).

FCN and core-shell nanostructures have been combined for oral gene delivery application. For instance, Nie et al. [96] developed a core-shell lipid/PEI-DNA nanoparticle to increase the low oral transfection efficiency often limited by the entrapment of cargos in the mucus layer and the gastrointestinal epithelial barrier. Surface-modified nanoparticles were produced in two microfluidic steps. Firstly, linear PEI was complexed with pDNA encoding glucagon-like peptide (GLP-1) using a CIJM, then coated with a lipid shell composed of DPPC and DMG-PEG through a MIVM (Figure 5B). The core-shell nanoparticles showed higher diffusivity and transport in the mucus layer of the gastrointestinal tract, mediating high levels of transfection efficiency in vitro and in vivo.

Recent studies also showed the potential of microfluidics to produce core/shell particles to combat the SARS-CoV-2 virus. Yang et al. [97] developed a core-shell structured COVID-19 mRNA vaccine (SW0123), which can be stored and transported under 4 °C using a commercial two-step microfluidic mixer. The core is composed of a cationic compound, SW-01, complexed with mRNA encoding the full-length SARS-CoV-2 Spike (S), and the shell is a mixture of ionized and non-ionized lipids. In vitro assays showed that the expression efficiency of EGFP-mRNA, a model molecule, was almost four times as high as that in the same cell line transfected with lipofectamine reagent. The authors showed that the core-shell nanoparticle facilitates vaccine uptake and demonstrates high colloidal stability. An in vivo desirable biodistribution pattern was also found for mRNA luciferase (mRNA Luc) and SARS-CoV-2 Spike (S). SW0123 showed strong immunogenicity and high antibody production levels, capable of neutralizing not only the wild-type SARS-CoV-2 but also the D614G and N501Y variants. SW0123 is currently being evaluated in a clinical trial (Phase I) in China [97].

### 2.6. Biomimetic Nanovesicles

Another alternative to produce mimicking natural membranes is by combining lipid nanostructures with molecules presented in mammalian membranes or extracellular vesicles. The same processes used to assemble lipids to form liposomes can be used for biomimetic nanovesicles assembly, in which the lipid alcoholic stream is hydrodynamically focused on the lateral aqueous streams containing the transmembrane proteins. In this case, the amphiphilic nature of the transmembrane proteins is the driving force for its insertion into the lipid bilayer.

Using this strategy, Molinaro et al. [102] produced a biomimetic vesicle named Leukosomes, the authors incorporated leukocyte membrane proteins into nanovesicles composed of DPPC, DOPC, and CHOL. This biomimetic nanostructure presented similar biological functions as the donor cells, and the microfluid process made possible the synthesis in one setup process in a bottom-up strategy (see Figure 5C). The same approach was used by Zinger et al. [104], who produced neurosomes using extracted proteins from hPSC-derived excitatory cortical neurons. The authors evaluated two different lipid compositions, DPPC/DOPC and DAP/DSPE-PEG2000/CHOL, with a protein:lipid mass ratio equal to 1:100. The authors found that the neurosomes (biomimetic human neural nanovesicles) presented a superior biological performance than simple nanovesicles (without neuronal transmembrane protein).

Another approach to form biomimetic vesicles is based on the combination of exosomes with conventional lipid nanostructures. Exosomes are a subtype of extracellular lipid nanostructures naturally released by cells. Depending on the cell type and cultivation culture, different compositions (proteins, RNA, DNA, and lipids) can be found in the exosomes. Exosomes can be used as a natural lipid system to be applied as a drug delivery system [136]. Similar to the nucleic acid-lipoplexes formation by microfluidic mixing, Yang et al. [105] mixed negatively charged exosomes containing exosomal RNA and cationic lipoplexes containing molecular beacons (CLP-MBs). The final lipoplex was further used for ultrafast and sensitive exosomal RNA detection for cancer diagnosis.

Different use of the exosome membrane (EM) or cancer cell membrane (CCM) was designed in a core/shell nanostructure. In this case, Liu et al. [103] developed a microfluidic system combining the hydrodynamic mixing and acoustic pulses (sonication) continuous process to promote the poly(lactic-*co*-glycolic acid) (PLGA) nanoparticle (PLGA-NPs) coating with EM or CCM (Figure 5D). As a control, conventional lipid-PLGA coated NPs are also produced. In terms of size, all three structures presented the same size. However, EM-PLGA NPs demonstrated superiority in evading the immune system, probably due to the protein composition.

## 3. Microfluidics for Lipid Micro-Sized Structures Synthesis

Liposomes are self-assembled phospholipid vesicles with great potential in fields ranging from targeted drug delivery to artificial cells. GUVs are vesicles that are >1 μm in diameter (most typically in the range of 10–30 μm). The large size of the GUVs and their low curvature enable us to analyze them individually through optical microscopes. Lipid vesicles can be composed of a single lipid component or mixtures (synthetic or natural lipids). Vesicles have also been made using many different surfactants besides phospholipids [137]. The production of a surfactant-free cell-vesicle has also been recently reported [138]. Due to their micrometer size, which is similar to the biological cells, GUV membranes are usually considered effective in studying protein–membrane interactions. Thus, they have been associated with proteins [139,140,141] or fragments from natural cell membranes [142]. In a bottom-up synthetic biology application, GUVs have been investigated as artificial biomimetic structures composed of synthetic and natural components. The manufactured cells may be applied for imitating cell behavior and acting as bioreactors.

The most popular methods for GUV production are lipid film hydration [143], the electroformation process [144], gel-assisted swelling [145], and the emulsion-based method [146]. These methods are critically limited by a lack of precision, resulting in highly non-uniform size distributions. The membrane features and encapsulated materials need to be well controlled [147]. In this context, other techniques, such as droplet-based microfluidics, have the advantages of precision control on the compartment size and structure with diameters from 500 nm to 500 μm and coefficient of variation around 2–3% [147,148]. For biological applications, these artificial cells can be formed by water-in-oil (W/O) [149], water-in-oil-in-water (W/O/W) [150], and water-in-oil-in-oil-in-water (W/O/O/W). Different microfluidic templates are used to produce artificial cells, for example, T-junction [149,151], flow-focusing [150,152], and coflowing [153,154]. Droplet-based microdevices allowed the construction of lipid-based vesicles by assembling a bilayer around the droplet exterior. In this system, the content of the droplet became the interior of the vesicle-based cell [155].

**Figure 6:**
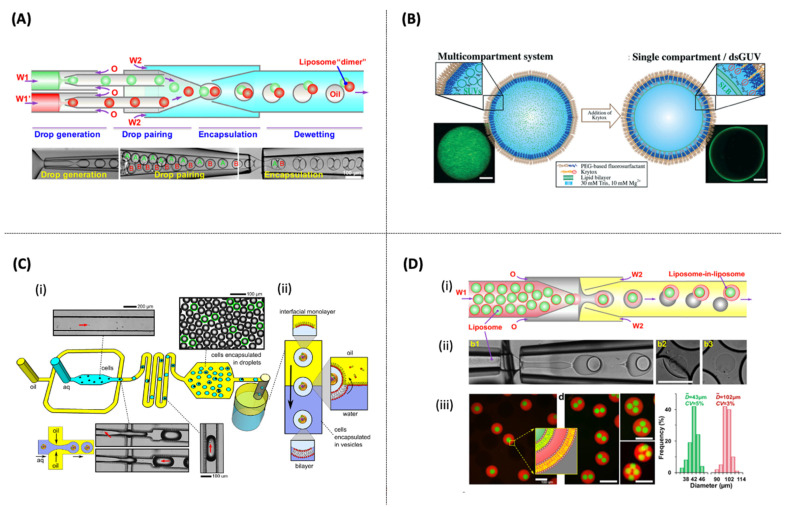
(**A**) Schematic device and images of double emulsions production with two distinct drops [156] (**B**) Charge-controlled microfluidic for the formation of a multicompartmental vesicle. dsGUVs: droplet-stabilized GUVs. Scale bars: 10 μm [157] (**C**) (**i**) Microfluidic device used to encapsulate cells in w/o droplets encased in a lipid monolayer. (**ii**) Schematic depicting the transformation of cells-in-droplets to cells-in-vesicles [80] (**D**) (**i,ii**) Microfluidic preparation of double emulsions with an inner liposome and the assembly of vesosomes from emulsion dewetting. (**iii**) Confocal images of the monodisperse vesosomes with one, two, three, and four inner liposomes. Size distribution of the inner and outer liposomes of the vesosomes. Scale bars: 100 μm [159].

Microfluidic-based fabrication may be applied to form uni- or multi-compartment vesicles [156,157] (Figure 6A). Cell microdroplets can act as independent picoliter reactors because they have been found in numerous applications in different scientific fields. For example, synthetic cells can be employed to design advanced drug-delivery systems and biomimetic cell behavior, enabling the encapsulation of hydrophobic/hydrophilic molecules, cells, and protein machinery [80,138,149,158].

Multicompartment synthetic cells have been created to encapsulate SUVs and LUVs in a high-throughput manner. Weiss et al. [149] showed that LUVs (100 nm) and GUVs (15 μm) and therapeutic cargo could be easily entrapped and released from the cell-like W/O droplet (around 40 µm in diameter). In this perspective, Haller et al. [157] also showed that SUVs (68 nm) could be easily entrapped into cell-like W/O droplets (around 25 in diameter) (Figure 6B). Weiss and coauthors [149] demonstrated that the encapsulated vesicles could be released into the physiological environment by changing their physicochemical and biochemical properties. The authors showed that the pico-injection technique might be coupled to the microfluidic device to load the cell-like compartment with transmembrane and cytoskeletal proteins.

In another study, Elani et al. [80] developed a W/O droplet microfluidic device to generate a hybrid cell-in-vesicle system composed of synthetic and cellular modules. These vesicle-based artificial cells are loaded with bioreactor modules such as biological cells (Figure 6C). The microsystem was validated for the encapsulation of bacterium and several eukaryotic cell lines, including Escherichia coli DH5α, BE colon carcinoma cells, HCT colon carcinoma epithelial cells, and Toledo B lymphocyte suspension cells.

Another potential application is shown by Yandrapalli et al. [138], who presented a (W/O/W) high-throughput microfluidic method to produce unilamellar vesicle sizes ranging from 10 to 130 µm. The GUV is composed of only neutral or charged lipids and without any surfactant or additive. The whole process was performed in physiological buffer conditions. The designed cell-like vesicle could efficiently encapsulate different cargos that are dispersible in aqueous solutions: pDNA (96%), SUVs 50 nm in diameter (94%), and fibroblast cells (75%). In the end, this system only showed low encapsulation efficiency (30%) for styrene microspheres, which are hydrophobic cargos.

The microfluidic production of a W/O/W double emulsion as templates for the formation of GUVs aiming at food and/or pharmaceutical applications was demonstrated by Michelon et al. [47]. Glass-capillary microfluidic devices were fabricated to create a genuinely three-dimensional flow, combining co-flow and flow-focusing. The GUVs were composed of food-grade phospholipids (soybean lecithin) and FDA-approved toxicological class III solvents. In this study, the challenge of microfluidic production of GUVs was the replacement of organic solvents potentially toxic for phospholipids dissolution commonly used such as toluene, chloroform, and hexane, by biocompatible green solvents, such as ethyl acetate and pentane. The challenge was overcome, and the results showed monodisperse and stable GUVs with diameters ranging between 100 and 180 μm and a coefficient of variation less than 6% [47].

Similarly, the microfluidic production of the GUVs based on the W/O/W double emulsion templates can also be achieved by 2D-PDMS microdevices, using a cross-junction with five input channels and one output channel [160]. In this process, the internal aqueous phase and the middle phospholipid phase focus on two perpendicular aqueous streams of the continuous phase. Thus, it is possible to produce stable and monodisperse W/O/W double emulsions in a single step.

Droplet microfluidics has also been reported for multicompartment vesicles production [156,159,161]. These multisomal systems, also known as vesosomes or vesicles-in-vesicles, have been reported as alternative systems with high potential as advanced drug delivery vehicles, bioreactors, and artificial cells. For the first time, Deng et al. [159] described a double emulsion method using a coflowing glass microcapillary device to prepare monodisperse vesosomes with one, two, three, and four inner liposomes (see Figure 6D). The size distribution of the inner and outer liposomes are 43 µm and 102 µm, respectively. It was demonstrated that vesosomes could be used as an in vitro transcription platform to synthesize RNA in the GUVs “nucleus” while mimicking the architecture of eukaryotic cells. Moreover, as a proof-of-concept, the transport of small fluorescent molecules from the inner liposomes to outer liposomes was achieved by inserting a membrane protein, melittin, which led to nanopore formation into the bilayers.

The microfluidic approach for generating giant liposomes has many advantages compared to traditional methods (gentle hydration and electroformation). Among these advantages, it is possible to cite the high-throughput production of monodisperse solutions, high encapsulation efficiency, and asymmetric lipid composition, typically found in biological cell membranes [162]. One disadvantage of emulsion-based technologies is that giant liposomes have been generated using organic solvents (e.g., chloroform, n-decane, and n-hexadecane) and surfactants/additives (PVA, PEG, and/or Pluronic F-68) dissolved in the phospholipid and aqueous phase, respectively. Thus, the challenge is to decrease the presence of nonbiological materials during the vesicle synthesis, for example, the amount of organic solvent in the organic layer and surfactant presence, without prejudicing liposome stability [138,162].

## 4. Additional Approaches for Sustained Release of Liposomes and Screening of Lipid Nanostructure Using Microfluidics

Beyond the classical synthesis of nano and micron-sized lipid structures, different microfluidic microdevices are being explored to study their biological performance in in vitro assays.

### 4.1. Microencapsulation of Liposomes for Drug Delivery Using Droplet-Based Microfluidics

Another challenge in lipid-based nanocarrier design and application is the development of strategies to in vivo sustained release. In general, conventional drug administration requires high dosages or repeated administration to achieve a therapeutic effect. These approaches can result in severe side effects, toxicity, lower overall efficacy, and patient compliance to the treatment [163,164]. In this context, hydrogels have been widely explored as a potential tool to encapsulate and release the desired payloads. The high-water content provides physical similarity to tissues and can give the hydrogels excellent biocompatibility. Hydrogel-based systems have shown to be a promising tool for sustained release of different payloads, such as small drugs, proteins, and even nanoparticles [165,166].

Different scaffolds and liposomes have been designed to improve the immobilization, release, and uptake of therapeutic genes [167,168,169,170,171] and drugs [166]. Due to their ease of injection and versatility, microgels have been used in a wide variety of drug-delivery applications. Microgels have been described as a preferable administration system compared to nano-sized carriers for specific tissue in which higher local doses are needed [172]. Taking advantage of micro and nano-scale systems, liposomes embedded in microgels have emerged as an attractive strategy to reduce the undesirable side-effects in drug delivery or tissue engineering applications. Moreover, the association between liposomes and polymeric matrices is a promising approach for minimizing the burst release caused by liposome instability. Recently, droplet-based microfluidics has been explored to form hydrogels in discrete volumes with characteristic dimensions in the range of micrometers and polydispersity values up to 2% [173,174]. From this perspective, microfluidics has been used to encapsulate within microgels small drugs [175], proteins [176,177], nanoparticles [81,178], liposomes [179,180,181,182], viral-vectors [183,184], and non-viral vectors [177]. In drug delivery, droplet-microfluidics have been investigated to associate liposomes and polymeric matrices to form liposomes-in-microgels [179,180,181,182,185] and lipobeads [186].

**Figure 7:**
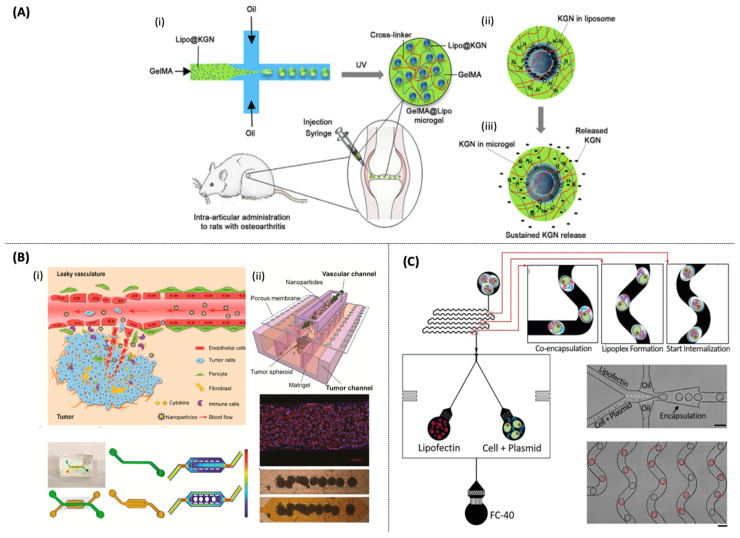
(**A**) (**i**,**ii**) Schematic illustration showing the (W/O) droplet-microfluidic fabrication of the KGN-loaded GelMA@Lipo hybrid microgel and its treatment in rat osteoarthritis via intraarticular injection. (**iii**) Schematic of the KGN release mechanism in GelMA@Lipo microgels [182]. (**B**) (**i**) Schematic of the in vivo tumor microenvironment consisting of a leaky vasculature and tumor tissues. (**ii**) Design and schematic illustration of a tumor-vasculature-on-a-chip (TVOC) [187] Scale bars: 100 μm. (**C**) Droplet microfluidics-based single-cell lipofection platform [188].

Yang et al. [182] developed one-step droplet-based microfluidics to immobilize kartogenin (KGN)-loaded liposomes (Lipo@KGN) within gelatin methacryloyl (GelMA) microgels (Figure 7A). Liposomes-in-GelMA-in-oil emulsions were generated using a microfluidic device wherein the resulting microgels-droplets are crosslinked under UV irradiation. Compared with Lipo@KGN, 250 nm in diameter, monodisperse GelMA@Lipo@KGN hybrid microgels, 100 μm in diameter, could extend the KGN release for over three weeks, promoting the chondrocyte differentiation of bone marrow mesenchymal stem cells. Moreover, the in vivo assay demonstrated that the hybrid microgel, with enhanced joint residence effect, could reinforce cartilage regeneration and inhibit osteoarthritis progression in mouse models.

Another study used a continuous two-step glass-capillary microfluidic technique to produce a multistage oral delivery system (liposome-in-microgel). The hybrid system is composed of a chitosan-coated insulin-loaded liposome (InsLip-CHT) and an enteric polymer, hydroxypropyl methylcellulose acetate succinate, which forms the microgel. In the first step, unilamellar Ins-loaded PEGylated liposomes were developed using a co-flow microfluidic device, 144 ± 23 nm, PDI of 0.1, ZP of −0.5 mV, and EE equal to 91%. Before the microencapsulation, InsLip was coated with CHT to improve their mucoadhesion, 363 nm, PDI of 0.3, and ZP of +23 mV. Then, the InsLip-CHT encapsulation into microgels was performed by W/O/W double emulsion using a flow-focusing microfluidic device forming a hybrid system of 19 μm in diameter. In vitro release assay showed Ins release starting above the pKa of microgel, pH 6.8, demonstrating efficient protection under gastric acidic conditions [179]. Collectively, these studies are excellent examples of how droplet-based microfluidics may be used to investigate the complex interaction between drug-loaded lipid-in-microgels and cells or local tissues.

In gene delivery, non-viral vectors have also been associated with hydrogels to form scaffold-gene non-viral carriers’ platforms to deliver genetic materials locally in vivo. The hydrogel reservoir system can increase transfection efficiency and allow long-term gene expression. Another advantage is that the controlled release of non-viral vectors may decrease their cytotoxicity and stability typically caused by the direct contact of cationic vectors and cell membranes. Even though gene-loaded liposome-in-hydrogel are often studied [167,168,169,170,171] in bulk, the application of non-viral vectors-in-microgel is still scarce in literature [177,185]. Within this perspective, droplet microfluidics may be a promising technique to be explored for building multistage systems for gene delivery. As in hydrogels, the local delivery of genes through a microgel could be a potential platform to increase the applicability of gene therapy in tissue regeneration and local therapies.

### 4.2. Microfluidic Platforms for Lipid-Based Nanocarriers Assays (Drug/Gene Delivery)

Microfluidic technology has also emerged as a tool to improve the complexity of the cellular environment. Microfluidic technology is well-known for many advantages, such as a quick and accurate response, portability, the capacity of handling minimal volumes, and low-cost process need. In drug/gene-delivery applications, all the interaction between drugs and therapeutic agents occurs within a microenvironment [189]. In contrast, conventional methods occur in a relatively large area, in which the desired cargos are randomly exposed in cells. In microfluidic cell culture devices, the large ratio between surface and volume may increase the cell uptake by changing the interaction between payloads and target cells [187]. Microfluidic tools have enabled strategies to study in vivo the complex nanocarrier transport and the resistance of cells to specific drugs [190,191,192].

In vitro microfluidic models have been developed to mimic better normal and unhealthy tissue than conventional 2D or 3D culture systems. These microfluidic cell culture platforms, known as organ-on-a-chip (OOC), are designed to mimic mechanical, biochemical, and functional properties similar to the in vivo microenvironment. Microdevices with these functions can operate dynamically, variating the fluid flows and mechanical cues that cells experience in organs and tissues, mimicking physiological functions [189]. This review described how microfluidic platforms had been investigated for lipid-based nanocarrier delivery studies in the last five years. Within this perspective, microfluidics is often used to elucidate the cellular behavior in vivo, focusing on the cell–cell, cell–matrix, and cell–lipid nanocarrier interactions. Recently, different OOCs have been developed to provide a rapid and reliable platform for evaluating pre-clinical drugs and nanomedicines, such as a blood–brain barrier-on-a-chip (BBB) [193], tumor-on-a-chip (TOC) [194], and tumor-vasculature-on-a-chip (TVOC) [187,195,196,197].

Wang et al. [187] designed a TVOC model combined with tumor spheroids in the extracellular matrix to mimic the enhanced permeability and retention (EPR) effect (Figure 7B). TVOC is used to investigate the liposome formulations, PEGylated liposome (PEG-Lip), and folic acid (FA)-Lip, for extravasation and tumor accumulation. The FA-Lip tumor level accumulation was verified in all biological assays. In the 2D monolayer model, FA-Lip promoted a higher cellular uptake than in the 3D tumor spheroid, while in TVOV and in vivo tumor models, nonsignificant accumulation levels were observed for the FA-Lip formulation. These results suggested that the dynamic TVOC model agreed better with animal models than the conventional static assays.

The same research group developed a TOC model that allows different-sized spheroid loading, formation, long-term cultivation, and drug evaluation. The TOC model provides a platform for screening the anticancer efficacy of liposomes (Lip). In this study, they investigated four different liposome formulations, including PTX-loaded PEGylated liposome (PEG-Lip), FA-Lip, cell-penetrating peptide TAT modified liposome (TAT-Lip), and FA and TAT commodified liposome (FA-TAT-Lip). When compared to all nanocarrier formulations, FA-TAT-Lip had the highest cytotoxicity and tumor accumulation. Adopting FA-TAT-Lip as the main nanocarrier, the tumor spheroid growth curve showed better tumor inhibition capability using lower flow rates. In addition, bigger tumor spheroids decreased the liposome binding and uptake efficiency. TOC model results corroborate better with the in vivo mouse assay than the 2D monolayer cell culture and 3D tumor spheroid models [194].

Despite all efforts in designing different types of OOC, few studies are related to the application of OOC in gene therapy studies [195,198,199]. However, in gene delivery, microfluidics is a potential technique to support gene-delivery procedures, wherein it is possible to find a dynamic system capable of controlling and monitoring “in real-time” the cell transfection assay [188,200,201]. This perspective explores microfluidics as a platform to improve the macromolecule intracellular delivery upon traditional methods [202,203,204,205].

In this perspective, Giupponi et al. [206] developed a microfluidic device for high-throughput screening of lipoplexes using two commercially sourced lipids, Lipofectamine 2000^®^ (Invitrogen, Thermo Fisher, Waltham, MA, USA) and FuGene^®^ 6 (Fugent LLC, Middleton, WI, USA) pEGFP-N1. The platform was used to evaluate simultaneously five transfection conditions generated by a chaotic serial dilution generator (lipoplex dilution from 100–0% with 25% steps). This analysis process helps to save large volumes of reagents while guaranteeing more precise control over cell behavior. The authors reported that this platform could be used to quantitatively assess the transfection efficiency and cytotoxicity in a spatio-temporally tunable microenvironment with a real-time investigation.

Li et al. [188] investigated the transfection of hard-to-transfect suspension cells via a single-cell approach using droplet-microfluidics. Droplets were used as microreactors to generate monodisperse lipoplexes (Lipofectamine + pcDNA3-EGFP plasmid) via chaotic mixing while encapsulating single cells (see Figure 7C). The transfection efficiency improved from 5 (bulk method) to 50% for all three studied suspension cell lines. Additionally, the authors found that the TP53BP1 gene could also be knockout via CRISPR9 in K562 cells.

Focusing on automated transfection process development, Raimes et al. [201] designed an automated perfused microfluidic device for long-term transfection culture assays. As a proof-of-concept, the authors showed that mouse embryonic stem cells were successfully transfected with a pDNA-GFP showing transfection efficiency of 34% compared to 17.2% (well plates transfection). The authors emphasized that the designed perfused microfluidic platform can be applied in future biological assays where long-term cell culturing is essential; for example, in deriving induced pluripotent stem cells (iPSC).

In general, microfluidic culture devices can explain how microenvironmental factors may influence tumor cell responses to anticancer therapies. Thus, microfluidic tools have enabled strategies toward whole OOC systems, which may transform the process of drug screening in the future. This new approach helps pre-clinical testing drug substances and toxicological studies while also providing more reliable pre-clinical pharmacokinetic and pharmacodynamic data.

## 5. Future Perspectives and Conclusions

Microfluidics has shown to be an emerging technology for the synthesis of nano and microstructured lipid-based systems. It is possible to develop bottom-up processes from simple liposomes to engineered and tailored nano or microstructures, such as core/shell, biomimetic vesicles, and giant liposomes. Microfluidic publications over the last five years have demonstrated the advances in microfluidics for lipid-based structure synthesis; however, successful market products in the pharmaceutic industry are still scarce. Although microfluidics production is still a challenge, it is important to highlight the fast development of lipid mRNA vaccines in 2020 against SARS-CoV-2. mRNA vaccines have demonstrated promising results regarding safety and efficacy. Pfizer/BioNTech and Moderna used a microfluidics-based production method to complex the (N1-methyl-pseudouridine) modified mRNA and LNPs [207,208]. It is important to highlight that the first publication about liposome formation using a microfluidic approach occurred less than two decades ago by Jahn et al. [64]. The development of lipid mRNA vaccines quickly demonstrates that microfluidics can be a promising technique for nanocarrier production.

Microfluidic platforms that allow a high throughput and flow rate capacity are already available in the market, such as the NanoAssemblr^®^ (Precision Nanosystems, Vancouver, BC, Canada) platforms. However, microfluidic technologies still need to be improved to expand production to make their industrial application feasible. In this sense, microfluidic platforms can be parallelized to replicate the conditions of single to multiple devices. Nevertheless, scalability remains challenging since it is difficult to guarantee the same operating conditions for hundreds of similar devices [21,32]. As an alternative to parallelization, new microfluidic platform designs can be developed to offer scale-independent production, and this is the case of the developed toroidal micromixer [32].

Before thinking about the industrial scale-up processes, the structural morphology of lipid nano and microstructures should be well elucidated. In this sense, current microfluidic designs can be improved using real-time characterization techniques to monitor any change during the lipid structure formation. Computational modeling and in silico experiments can also bring us trenchant insights into nanoformulation designing and particle interactions. In this sense, recently studies have used advanced on-chip characterization techniques such as DLS, confocal Raman microscopy, X-ray absorption spectroscopy [209], SAXS [210], and SANS [211] to understand the relationship between microfluidic processes and nanoformulation. Recently, microfluidics has also been associated with artificial intelligence technologies to process a large amount of obtained data in nanomedicine and material synthesis [212].

Although many studies aim to develop lipid systems, microfluidic production is still a technological challenge. This review shows that microfluidics’ development of a biomedical solution involves different research fields such as chemical, biology, pharmacy, and engineering. The development of new solutions may often require further steps for pre-clinical tests such as mimicked systems to simulate in vitro delivery and the development of novel microfluidic devices for drug/gene screening. Searching for newly designed lipids is also necessary while combining specific ligands and biomaterials with other materials. It is expected that more effective and scalable self-assembly strategies in this multidisciplinary field will lead to more nano and micro lipid structures emerging in the next decade.

## Figures and Tables

**Table 1:** Lipid-based nanocarrier systems (liposome, lipoplex, cationic LNP (cLNP), and ionizable LNP (iLNP)) produced by microfluidics for drug and gene delivery.

Type of Nanocarrier	Nanocarrier Composition	Therapeutics	Microfluidic Device Type	Potential Application	Ref.
Liposome	PC and CHOL	Propofol	SHM *	Anesthesia	[68]
	PC, DMPC, DPPC, CHOL, PS, and DSPC	Insulin, BSA, or OVA	SHM *	-	[69]
	HSPC, CHOL, and DSPE-PEG2000	DOX	N/A *	Cancer: MDA-MB231cells and xenograft model bearing MDA-MB231tumor	[82]
	PC, DMPC, DPPC, and DSPC	Glipizide and metformin	SHM *	Diabetes	[83]
	DMPC, DPPC, and DSPC	Cisplatin and Curcumin	SHM *	Cancer: EMT6 and B16F10 cells/and xenograft model bearing EMT6 and B16F10 tumor	[84]
	DSPC, CHOL, and DSPE-PEG2000	DOX and UMB	5-Input Chip **	Cancer: MCF-7, MDA-MB231, and BT-473 cells	[85]
	DMPC, DSPE-PEG, and CHOL. Ligands: DSPE-PEG-TAT and DSPE-PEG-Folate	-	HFF device	Cancer: SKOV3 and MCF-7 cells and 3D tumor spheroids/and xenograft model bearing SKOV3 tumor	[86]
Lipoplexes	cationic lipid DOTAP, EPC, and DOPE	pEGFP-N1	HFF device	PC3 cells	[87,88]
	cationic lipid (DOTAP, DDA, DC-CHOL, DMTAP, DSTAP or DOBAQ), DOPE or DSPC, CHOL, and DMG-PEG2000	SAM encoding rabies virus glycoprotein (RVG)	Y-shape SHM *	Prophylactic vaccine:BHK cells and BALB/c mice	[72]
	cationic lipid DOTAP, DC-CHOL, DOPE, CHOL, and DOPE-PEG	pGL3	Y-shape SHM *	HEK-293, HaCaT, N/TERT, and CaSki cells	[89]
	pH-sensitive cationic lipid YSK05, chol, and PEG-DMG	siFVII	Baffle mixer device	ICR mice liver tissues: hepatocytes delivery and FVII gene-silencing activity.	[90]
iLNP and cLNP	Ionizable lipid MC3 or cationic lipids DOTAP and DDAB, HSPC or DSPC, CHOL, and DMG-PEG2000 or DSPE-PEG2000	PolyA, ssDNA or mFLuc	Y-shape SHM *	N/A	[70]
	Ionizable lipid (MC3, KC2, DODMA) or cationic lipid (DOBAQ, DOTMA, DOTAP), DSPC, DMG-PEG2k, and CHOL	mRNAs: mFLuc, mEGFP, and mCherry	N/A	Retinal degeneration: BALB/c mice	[91]
iLNP	Ionizable lipid (C12−200), DOPE or DSPC, CHOL, and lipid-PEG	siFVII or mLuc	SHM parallelized device	HeLa cells and C57BL/6 mice	[21]
	Ionizable lipids MC3 or KC2, DLinDAP or DLinDMA, CHOL, DOPE, DOPC, SOPC, DLinPC, DPoPC or DSPC, and DMG-PEG	pDNAs: pEGFP or pFLuc	T-junction mixer	HeLa, HepG2, Hep3B, PC12, and MCF7 cells (in vitro) and leghorn chicken embryos (in vivo)	[92]
	ATX ionizable amino lipids, CHOL, DSPC, and DMG-PEG	pWRG/c7d11	N/A*	Prophylactic Andes and Zika virus vaccine: Vero cells, rabbits, and nonhuman primates	[93]
	Ionizable lipid KC2, CHOL, DSPC, and DMG-PEG	mFLuc or mcDNA	T-junction mixer	N/A	[94]

* NanoAssemblr Benchtop ™ (Precision Nanosystems, Vancouver, BC, Canada). ** Automated Dolomite microfluidic system (Dolomite, Royston, UK).

**Table 2:** Nanocarrier systems (core/shell NPs and exosomes) produced by microfluidics for drug and gene delivery.

Type of Nanocarrier	Nanocarrier Composition	Therapeutics	Microfluidic Device Type	Potential Application	Ref.
	CORE: PCL-PEI/SHELL: CHOL, DSPE-PEG2000, and DOPE	siEGFR	Three-stage microfluidic chip (MiTASChip Ltd., Jiangsu, China)	Cancer: PC3 cells and xenograft model bearing PC-3 tumor	[95]
	CORE: PEI/SHELL: CHOL, DPPC, and DMG-PEG	pGLP-1	CIJM and MIVM	Oral delivery type II diabetes-293T, A549, HepG2, HeLa cells, and BALB/c mice	[96]
	CORE: Cationic material (SW-01)/SHELL: ionizable lipid, DOPE, and PEG-lipid	mRNAs: mEGFP and mSARS-CoV-2 Spike (S) (in vitro)/mLuc (in vivo)	Two-step microfluidic mixer (Inano D, Micro&Nano Technology Inc., China)	Prophylactic COVID vaccine: DC 2.4, HEK-293 T cells, and BALB/c mice	[97]
	CORE: PLGA/SHELL: Lecithin and DSPE-PEG 2000	Sorafenib	Borosilicate glass capillaries	Cancer: MDA-MB231, PC3-MM2, and HT29-MTX cells	[98]
	CORE: PLGA/SHELL: DOTAP, DOPE, CHOL, DPPC, and DSPE-PEG	-	Two-stage microfluidic device	HUVEC cells and BALB/c mice	[99]
	CORE: PLGA and CPP-SA/SHELL: DPPC, DSPE-PEG, and CHOL	Ketamine and hydromorphone	Two-stage microfluidic device	Intractable neuropathic pain: Chronic constriction injury (CCI)-rats	[100]
	CORE: PLGA/SHELL: Lecithin and DSPE-PEG	Rifampicin	MIVM and herringbone-patterned MIVM	Tuberculosis	[101]
BIOMIMETIC VESICLES	LIPIDS: DPPC, DOPC and CHOL/PROTEIN: leukocyte membrane proteins	-	N/A *	J774 macrophages	[102]
	CORE: PLGA/SHELL: cancer cell or exosome membranes or lipids (DPPC, CHOL, and DSPE-PEG)	-	Two-stage microfluidic device	Cancer: A549, MDA-MB-231, RAW 264.7 cells, and xenograft model bearing A549 and MDA-MB-231tumors	[103]
	LIPIDS: (1) DPPC, DOPC, and CHOL and (2) DAP, DSPE-PEG2000, and CHOL/PROTEIN: hPSC-derived excitatory cortical neurons	-	N/A *	Human pluripotent stem cells (hPSCs) and trigeminal ganglion of C57BL/6mice	[104]
	LIPIDS: DOTMA, CHOL, TPGS	Molecular beacons: TPGS exosomal RNA FAM-miR-21 MBs and Cy5-TTF-1 MBs	Layer-by-layer micromixer biochip	Cancer: A549 NSCLC and BEAS-2B cells	[105]

* NanoAssemblr Benchtop ™ (Precision Nanosystems, Vancouver, BC, Canada).

## Abbreviations

DOPE	1,2-dioleoyl-sn-glycero-3-phosphoethanolamine
PCL-PEI	polyethylenimine-graft-polycaprolactone
CHOL	cholesterol
DSPE-PEG2000	1,2-distearoyl-sn-glycero-3-phosphoethanolamine-*N*-[methoxy (polyethylene glycol)]-2000
PEI	polyethylenimine
DPPC	1,2-dipalmitoyl-sn-glycero-3- phosphocholine
DMG-PEG	1,2-dimyristoyl-rac-glycero-3-methoxy poly (ethylene glycol)-2000
PLGA	poly (lactic-*co*- glycolic acid)
DOTAP	1,2-dioleoyl-3-trimethylammonium-propane
CPP-SA	poly-carboxyphenoxy propane *co*-sebacic acid
PEG	polyethylene glycol
DC-Chol	3-β-[*N*-(*N_0_*, *N_0_*-dimethylaminoethane)-carbamoyl])-cholesterol
DOPC	1,2-dioleoyl-sn-glycero-3-phosphocholine
EPC	egg phosphatidylcholine
DSPC	1,2-distearoyl-sn-glycero-3-phosphocholine
PC	phosphatidylcholine
DMPC	1,2-dimyristoyl-sn-gly- cero-3-phosphocholine
DPPC	1,2-dipalmitoyl-sn-glycero-3-phos-phocholine
PS	l-α-phosphatidylserine
HSPC	hydrogenated soy l- α-phosphatidylcholine (HSPC)
KC2	2,2-dilinoleyl-4-(2-dimethylaminoethyl)-[1,3]-dioxolane
PEG-DMPE	1,2-dimyristoyl-sn-glycero-3-phosphoethanolamine-*N*-[methoxy(polyethylene glycol)-2000]
DOPE-PEG	1,2-dioleoyl-sn-glycero-3-phosphoethanolamine-*N*-[amino(polyethylene glycol)]-2000
DLinDAP	1,2-dilinoleoyl-3-dimethylaminopropane
DLinDMA	1,2-dilinoleyloxy-3-dimethylaminopropane
MC3	heptatriaconta-6,9,28,31-tetraen-19-yl 4-(dimethylamino)butanoate
SOPC	1-stearoyl-2-oleoyl-sn-glycero-3-phosphocholine
DDAB	dimethyldioctadecylammonium
DODMA	1,2-dioleyloxy-3-dimethylaminopropane
DOBAQ	*N*-(4-carboxybenzyl)-*N,N*-dimethyl-2,3-bis(oleoyloxy)propan-1-aminium
DOTMA	1,2-di-O-octadecenyl-3-trimethylammonium propane
DDA	dimethyldioctadecylammonium
DMTAP	1,2-dimyristoyl-3-trimethylammonium-propane
DSTAP	1,2-stearoyl-3-trimethylammonium-propane
DLinPC	1,2-dilinoleoyl-sn-glycero-3-phosphorylcholine
DPoPC	1-palmitoyl,2-oleoyl-sn-glycero-3-phosphorylcholine
TPGS	d-α-tocopherol polyethylene glycol 1000 succinate
DAP	1,2-dipalmitoyl-3-dimethylammonium-propane
HFF	hydrodynamic flow-focusing
CA-M	chaotic advection-based micromixer
SHM	staggered herringbone micromixer
MIVM	multi-inlet vortex mixer
CIJM	confined impingement jet mixer
